# Using a Matrix Retainer in Plastic Filling

**Published:** 1902-05-15

**Authors:** R. G. Hutchinson


### USING A MATRIX RETAINER IN PLASTIC FILLING.

BY R. G. HUTCHINSON.

Plastic fillings are often imperfectly made because of the imperfect adaptation of the material at the cervical borders. There is either an excess or deficiency, or it is imperfectly condensed. I have had the greatest pleasure in using a matrix of the Ivory pattern, which can be drawn close up to the cervical margins by the No. 1 Ivory matrix retainer.

It is a common thing for plastic fillings to fail at the cervical margin. I have many times found extensive inflammation, with loss of tissue, resulting from a large excess of amalgam projecting against the gum, and in cases too where there was evidence of care and skillful manipulation manifested in the rest of the filling.

Such a condition need not exist if the operator is careful to trim the filling with suitable instruments before it has had time to set. The use of this matrix, however, renders the operation much more sure and simple, and the contour may be built up more perfectly and left in contact with the adjoining tooth, as the band is so thin that no appreciable space is left.

I consider it advisable after removing the matrix to pass a small quantity of dental fiber through so that any loose bits or possible excess that may have been forced through, will be removed.

Without a matrix it is a most difficult matter to make a perfect cement filling, in, for instance, an anterior approximal cavity involving the grinding surface of a molar. If you use a quick setting cement and pay proper attention to the cervical margin, the chances are that you cannot give the contour and form you desire, or perhaps more commonly the contour and grinding surface receive the most attention and the cervical margin is left to suffer.

There may be a surplus of material bulging out of the cavity, or more likely, a space left unfilled, where recurrent caries will begin almost immediately. If you succeed in making a perfect filling by burnishing down the cement while soft at the cervical margin, unless the rubber dam is on, your instrument will conduct the moisture from the gum margin and disintegration of the cement will result at that point on account of its having become wet while setting. When this matrix is applied, the gum margin is held away from the cavity, giving full access to the cavity margin and excluding moisture.

Another point that is gained is the ability to trim the margin perfectly after the matrix is on the tooth. I have often found that where I thought the margins perfect, there were thin edges which appeared only after adjusting the matrix, and the failure to remove these would undoubtedly have made the fillings less serviceable. This applies more especially to cases where the cavity extends so far under the gum margin that the rubber dam cannot be applied and there is considerable hemorrhage.

In cases of badly broken down third molars in the lower jaw, where the gum has grown into the cavity, it is possible to hold this growth out, exposing the entire cavity, and restore the tooth perfectly with any plastic material desired.

I do not advocate the use of this matrix for gold fillings, but have, upon several occasions, where there was not time to make a separation, been successful in that direction. The matrix can be applied to any approximal surface of any of the molars or bicuspids of either jaw. If it should not draw in close to the neck of the tooth, it can be forced up by wedging in a pellet of cotton.

There is no doubt in my mind that many failures could have been averted by the use of a good matrix, for since using this one I have had much better results than before.

Of course perfect results will not be obtained unless care is taken to carry the filling material up to the cervical margin against the matrix. If a small amount is forced through it can be easily removed after the filling has set and the band has been taken off the tooth. —*Items of Interest.*

